# Regional differences in the inflammatory and heat shock response in glia: implications for ALS

**DOI:** 10.1007/s12192-019-01005-y

**Published:** 2019-06-05

**Authors:** Benjamin E. Clarke, Rebecca San Gil, Jing Yip, Bernadett Kalmar, Linda Greensmith

**Affiliations:** 1 Department of Neuromuscular Diseases, University College London (UCL) Queen Square Institute of Neurology, London, WC1N 3BG UK; 2 MRC Centre for Neuromuscular Disease, London, WC1N 3BG UK; 3 Illawarra Health and Medical Research Institute, School of Biological Sciences, University of Wollongong, Northfields Ave, Wollongong, 2522 Australia

**Keywords:** Astroglia, Microglia, Inflammation, NF-κB, iNOS, NO, Heat shock response, Heat shock protein 70, ALS

## Abstract

Preferential neuronal vulnerability is characteristic of several neurodegenerative diseases including the motor neuron disease amyotrophic lateral sclerosis (ALS). It is well established that glia play a critical role in ALS, but it is unknown whether regional differences in the ability of glia to support motor neurons contribute to the specific pattern of neuronal degeneration. In this study, using primary mixed glial cultures from different mouse CNS regions (spinal cord and cortex), we examined whether regional differences exist in key glial pathways that contribute to, or protect against, motor neuron degeneration. Specifically, we examined the NF-κB-mediated inflammatory pathway and the cytoprotective heat shock response (HSR). Glial cultures were treated with pro-inflammatory stimuli, tumour necrosis factor-ɑ/lipopolysaccharide or heat stressed to stimulate the inflammatory and HSR respectively. We found that spinal cord glia expressed more iNOS and produced more NO compared to cortical glia in response to inflammatory stimuli. Intriguingly, we found that expression of ALS-causing SOD1^G93A^ did not elevate the levels of NO in spinal cord glia. However, activation of the stress-responsive HSR was attenuated in SOD1^G93A^ cultures, with a reduced Hsp70 induction in response to stressful stimuli. Exposure of spinal cord glia to heat shock in combination with inflammatory stimuli reduced the activation of the inflammatory response. The results of this study suggest that impaired heat shock response in SOD1^G93A^ glia may contribute to the exacerbated inflammatory reactions observed in ALS mice.

**Graphical abstract:**
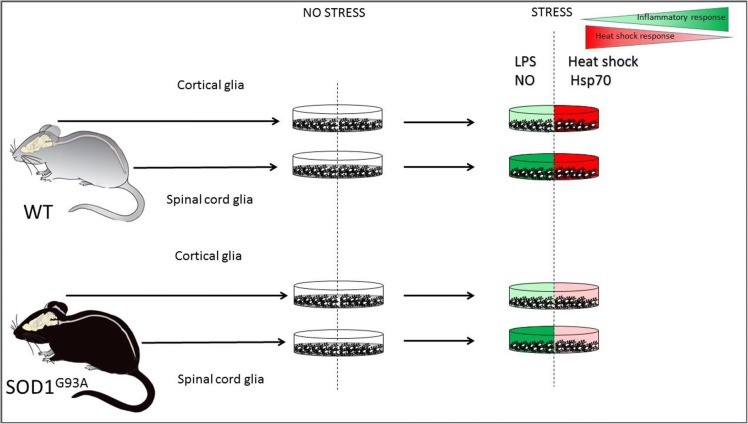
Mixed primary glial cultures were established from cortical and spinal cord regions of wild-type mice and mice expressing ALS–causing mutant human SOD1 and the inflammatory and heat shock responses were investigated in these cultures. In the absence of stress, all cultures appeared to have similar cellular composition, levels of inflammatory mediators and similar expression level of heat shock proteins. When stimulated, spinal cord glia were more reactive and activated the inflammatory pathway more readily than cortical glia; this response was similar in wild-type and SOD1^G93A^ glial cultures. Although the heat shock response was similar in spinal cord and cortical glial, in SOD1^G93A^ expressing glia from both the spinal cord and cortex, the induction of heat shock response was diminished. This impaired heat shock response in SOD1^G93A^ glia may therefore contribute to the exacerbated inflammatory reactions observed in ALS mice.

Mixed primary glial cultures were established from cortical and spinal cord regions of wild-type mice and mice expressing ALS–causing mutant human SOD1 and the inflammatory and heat shock responses were investigated in these cultures. In the absence of stress, all cultures appeared to have similar cellular composition, levels of inflammatory mediators and similar expression level of heat shock proteins. When stimulated, spinal cord glia were more reactive and activated the inflammatory pathway more readily than cortical glia; this response was similar in wild-type and SOD1^G93A^ glial cultures. Although the heat shock response was similar in spinal cord and cortical glial, in SOD1^G93A^ expressing glia from both the spinal cord and cortex, the induction of heat shock response was diminished. This impaired heat shock response in SOD1^G93A^ glia may therefore contribute to the exacerbated inflammatory reactions observed in ALS mice.

## Introduction

The majority of cells in the CNS are glial cells, including astroglia and oligodendrocytes as well as microglia, which are derived from haematopoietic cells that infiltrate the CNS during development and act as the resident macrophages of the CNS. Together, glial cells play crucial roles in the maintenance of normal neuronal function, providing metabolic support and regulating synaptic networks, while also conferring protection under conditions of cellular stress (Khakh and Sofroniew 2015; Marina et al. 2018).

Unsurprisingly, for a cellular component so abundant in the CNS, in neurodegenerative conditions such as amyotrophic lateral sclerosis (ALS), glial cells are now known to play a critical role in the progression of disease. Glia actively contribute towards motor neuron death in ALS by propagating a toxic pro-inflammatory environment for motor neurons (Chen et al. 2018; Haidet-Phillips et al. 2011). In mutant SOD1 mouse models of ALS that recapitulate several aspects of the disease including neuroinflammation, motor neuron loss and reduced lifespan, deletion of the mutant gene from either astroglia or microglia slows the progression of disease (Boillee et al. 2006; Yamanaka et al. 2008). Furthermore, mutant SOD1–expressing astroglia and microglia are toxic to motor neurons in co-culture (Bilsland et al. 2008; Frakes et al. 2014; Nagai et al. 2007). Although the precise mechanisms of this non-cell autonomous motor neuron death remain unknown, the release of an unidentified toxic factor (Re et al. 2014), loss of trophic support mechanisms (Ferraiuolo et al. 2011; Rothstein et al. 1995), dysregulation of stress-inducible responses and/or dysregulation of the immune response (Puentes et al. 2016) have all been reported to be involved.

Glia exist as regional subtypes based on differential transcriptomes and proteomes (Barbin et al. 1988; Zhang and Barres 2010; Molofsky et al. 2014; Hochstim et al. 2008; Grabert et al. 2016). The development of glial subtypes is dependent on distinct molecular signatures (Chai et al. 2017; Ben Haim and Rowitch 2017; de Haas et al. 2008). Not only do spinal cord and cortical glial cultures differ in their abundance of astroglial and microglial subtypes, they also display marked functional differences (Barbin et al. 1988; Grabert et al. 2016; Schitine et al. 2015). For example, cortical glia promote neuronal dendrite development far more than spinal cord glia (Leroux and Reh 1994) and the glutamate transporter GLT-1 is expressed 10-fold higher in cortical astroglia than in spinal cord astroglia (Regan et al. 2007). Furthermore, differences between cortical and spinal cord glia in potassium buffering results in marked differences in synaptic regulation (Oberheim et al. 2012). As neurodegenerative diseases are characterised by the loss of specific neuronal populations, surrounding glial populations may display functional differences in vulnerable regions of the CNS, contributing to disease in a region-specific manner. It is possible that regional differences in glia play a role in the selective degeneration of motor neurons in the SOD1^G93A^ mouse model of ALS, in which motor neuron death occurs earlier and to the greatest extent in the spinal cord (Leichsenring et al. 2006).

One of the major roles of glial cells in the CNS is to act as part of the immune system. Although microglia are the resident immune cells of the CNS, astroglia also play a role in both mediating and responding to microglial activation in response to cell damage and infection (Liddelow and Barres 2017; Liddelow et al. 2017; Ouali Alami et al. 2018). Chronic neuroinflammation is a major pathological hallmark of ALS and is driven by the pro-inflammatory activities of microglia and astroglia in the CNS (Philips and Robberecht 2011; Hooten et al. 2015). Activation of microglia and astroglia in response to inflammatory stimuli such as acute traumatic injury and neurodegenerative conditions can lead to the activation of irreversible self–destructing pathways, mediated by the inflammatory transcription factor NF-κB (Akama and Van Eldik 2000; Birck et al. 2016). This process involves the activation of inflammatory mediators such as inducible nitric oxide synthase (iNOS), producing highly oxidative nitric oxide (NO). Excessive NO production in the CNS contributes to neuronal damage leading to neuronal loss in ALS and other neurodegenerative conditions (Yuste et al. 2015; Drechsel et al. 2012).

Glial cells also confer protection to neurons through the protective intracellular heat shock response (HSR) (Gleixner et al. 2016; Xia et al. 2016). The HSR is activated under conditions of cellular stress and leads to an increase in the synthesis of several stress-inducible chaperones called heat shock proteins (Hsps) in the cytosol and in subcellular organelles such as the ER and mitochondria (Kalmar and Greensmith 2017). The main effector of the HSR is the cytosolic Hsp70, which is highly inducible in some neuronal populations as well as glial cells under a wide range of stressful stimuli (Manzerra and Brown 1996; Magrane et al. 2004). Hsp70 has been shown to be protective to glia and indirectly to neurons (Xu et al. 2010). Interestingly, spinal cord motor neurons have a surprisingly high threshold for the induction of the HSR, possibly due to a reliance on the supply of Hsps from glia (Guzhova et al. 2001; Batulan et al. 2003; Robinson et al. 2005). Under normal physiological conditions, the inflammatory and HSR pathways counterbalance each other, with Hsp70 acting as an inhibitor of the NF-κB inflammatory pathway (Kim et al. 2015; Kacimi and Yenari 2015; Feinstein et al. 1996).

In this study, we compared the stress responses of primary cortical and spinal cord mixed glial cultures obtained from neonatal mice in response to exposure to heat shock and inflammatory stimuli. This model enables the study of the complex interactions between different glial populations, providing a closer in vitro representation of the cortex or spinal cord than pure astroglial or microglial cultures. Hsp70 expression levels were used as a marker of activation of the HSR and measurements of NO release and iNOS expression were used to assess activation of the NF-κB-mediated inflammatory pathway. Finally, we explored whether regional differences between cortical and spinal cord glia are altered in cultures obtained from mice expressing mutant SOD1^G93A^, a model of mutant SOD1–induced ALS in which inflammatory and neuronal damage occurs to a greater extent in the spinal cord than in the cortex. The results of our study provide evidence for the regional specificity of glial stress responses and suggest that impairments in the HSR of SOD1^G93A^ glia might contribute to the excessive neuroinflammation that is known to play a role in motor neuron degeneration in ALS.

## Results

**Fig. 1:**
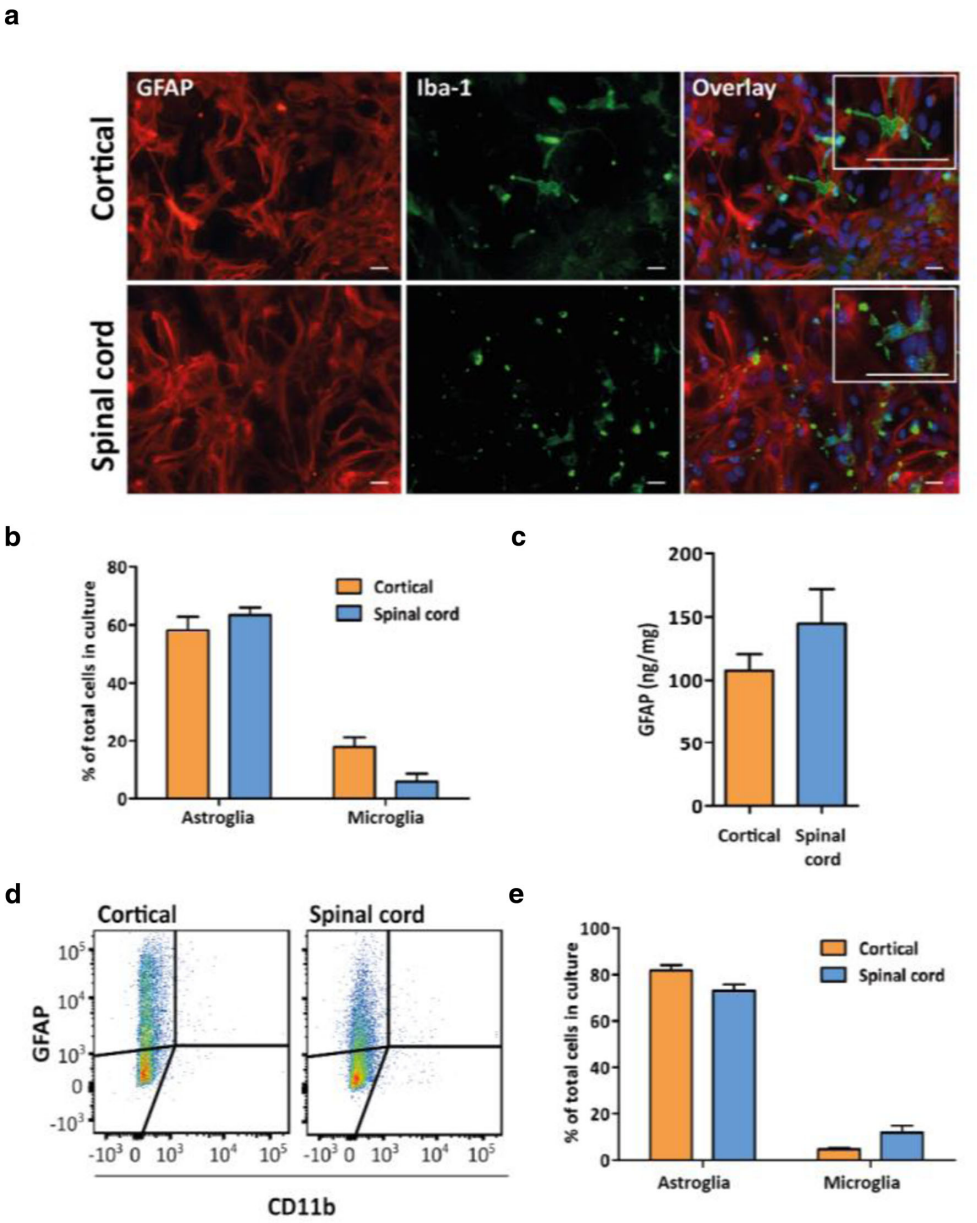
Characterisation of glial cultures reveals that cultures from the cortex and spinal cord have a similar cellular composition. **a** Mixed glial cultures at 12 days in vitro stained for glial markers GFAP (red, astrocytes) and iba-1 (green, microglia). **b** Quantification of GFAP and iba-1 expressing cells in cortical and spinal cord mixed glial cultures. **c** ELISA assay for GFAP expression in cortical and spinal mixed glial cells. **d** Characterisation of cortical and spinal cord mixed glial cultures performed by FACS analysis of GFAP-positive (astrocytes, Y axis) and CD11b (microglia, X axis)-positive cells. **e** Quantification for FACS analysis of cortical and spinal cord mixed glial cultures. Error bars = S.E.M. Scale bar, 20 μm

The stress-induced responses of glia from different regions of the CNS were examined in vitro in mixed glial cultures established from the spinal cord and cortex firstly of neonatal wild-type (WT) mice and, subsequently, in glia derived from SOD1^G93A^ mice that model ALS. In this study, we examined two key stress responses of glial cells: the response to (i) inflammatory mediators and (ii) the HSR. To ensure that our assessments reflected true regional differences in glial function and were not the result of different cellular composition of cultures from different CNS regions, we first established the cellular composition of the mixed glial cultures derived from the cortex and spinal cord of wild-type mice. The analysis of the composition of cortical- and spinal cord–derived mixed glial cultures included immunofluorescence, ELISA and FACS analysis using GFAP as an astroglial marker and Iba1 or CD11b as a microglial marker (Fig. 1).

### Mixed glial cultures from the cortex and spinal cord consist of similar proportions of astroglia and microglia

Analysis of immunofluorescence staining for cell-specific markers demonstrated comparable proportions of GFAP-positive cells and Iba-1-positive cells in cortical and spinal cord cultures (Fig. 1a, b). The total GFAP content in whole cell lysates of both cultures determined by ELISA supported the immunofluorescence findings and showed no difference in GFAP levels between cortical and spinal cord mixed glial cultures (Fig. 1c). Furthermore, FACS analysis of cells double labelled with GFAP and CD11b demonstrated that there were no significant differences between the proportion of astroglia (~ 80%) and microglia (~ 10–20%) in cortical and spinal cord cultures (Fig. 1d, e). Therefore, in the experiments that follow, any differences in the stress responses of glial cultures established from the spinal cord and cortex were not due to a difference in the cellular composition of the cultures.

### Spinal cord–derived glial cultures produce more NO and express higher levels of iNOS in response to inflammatory stimuli than cortical glia

**Fig. 2:**
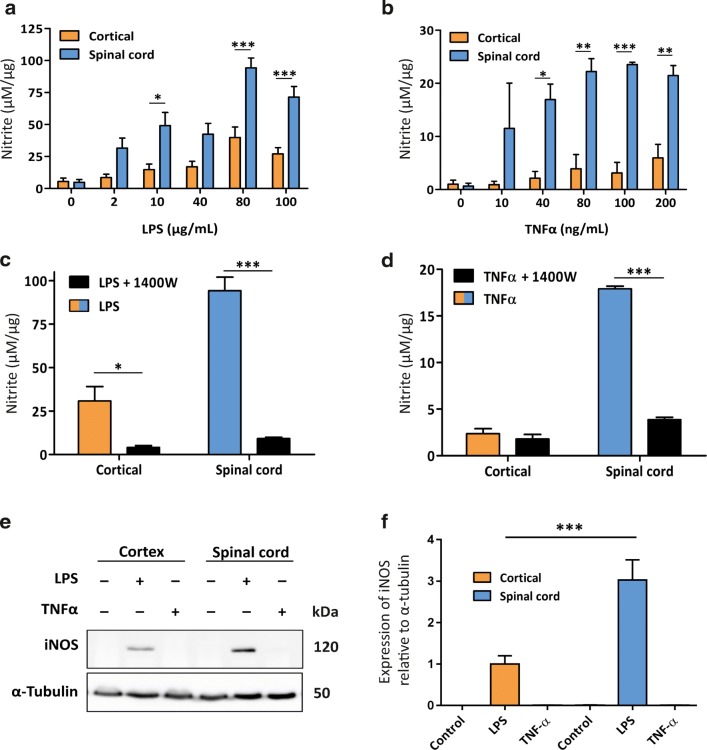
Elevated NO production and iNOS expression in spinal cord–derived glial cultures compared to cortical glia in response to inflammatory stimuli. Nitrite/nitrate production in response to increasing concentrations of LPS (**a**) or TNF-ɑ (**b**), and the specific iNOS inhibitor 1400W (**c**, **d**) was measured using a Griess assay. **e** Immunoblot for iNOS expression in cortical and spinal cord mixed glial cultures exposed to LPS or TNF-ɑ. f Quantification of immunoblots for iNOS in cortical and spinal cord mixed glial cultures exposed to LPS or TNF-ɑ. Error bars = S.E.M.

NO was as used as marker of the glial inflammatory response, based on previous studies of glial cells in neurodegenerative conditions, including Parkinson’s disease and ALS (Drechsel et al. 2012; Tripathy et al. 2015). We compared the ability of cortical and spinal cord mixed glial cultures to produce NO in response to increasing concentrations of pro-inflammatory stimuli: the endogenous pro-inflammatory cytokine TNF-ɑ and the bacterial endotoxin LPS. Although baseline NO production in cortical and spinal cord-derived glial cells was very similar, spinal cord glia produced significantly higher levels of nitrite and nitrate than cortical glia when stimulated with increasing concentrations of LPS or TNF-ɑ (Fig. 2a, b). The data in Fig. 2a, b demonstrates that the CNS region from which the glial cultures were derived significantly affected the concentration of nitrite in response to each inflammatory mediator, with spinal cord glial cultures generating a 2–4-fold greater concentration of nitrite compared to cortical glia after LPS treatment and 5–12-fold greater concentration after TNF-α treatment.

Next, we sought to determine the source of NO production in these mixed glial cultures. NO can be produced by several different enzymes including endothelial nitric oxide synthase (eNOS), neuronal nitric oxide synthase (nNOS) and iNOS (Yuste et al. 2015). To determine the source of the NO synthesis, LPS- and TNF-α-treated mixed glial cultures were pre-treated with 1400W, a compound that has previously been shown to selectively and irreversibly inhibit iNOS activity in primary cultures at a range of concentrations (5–400 μM) (Garvey et al. 1997; Saura et al. 2005). The iNOS-specific inhibitor, 1400W, completely inhibited NO production in both cortical- and spinal cord–derived mixed glial cultures (Fig. 2c, d), confirming that the NO measured in the media in our cultures was a product of iNOS activity. In line with the increased NO production, there was a significant, threefold increase in iNOS expression in LPS-treated spinal cord glia compared to cortical glia (Fig. 2e, f). TNF-α treatment did not lead to detectable iNOS expression. The low concentration of NO detected after TNF-α treatment could be the consequence of lower iNOS expression, which may be below the detection limit of the iNOS antibody with the immunoblotting procedure used in this work.

Thus, although under baseline conditions cortical and spinal cord glial cultures produced very similar, low levels of NO and no detectable iNOS, spinal cord glia more readily increased iNOS expression and produced higher levels of NO than cortical glia when exposed to the same level of inflammatory stimuli. These findings suggest that spinal cord glia have a lower threshold for the activation of the NFκ-B-mediated inflammatory pathway and a stronger inflammatory response than cortical glia.

### Expression of SOD1^G93A^ in glia does not result in elevated NO production or higher iNOS expression

**Fig. 3:**
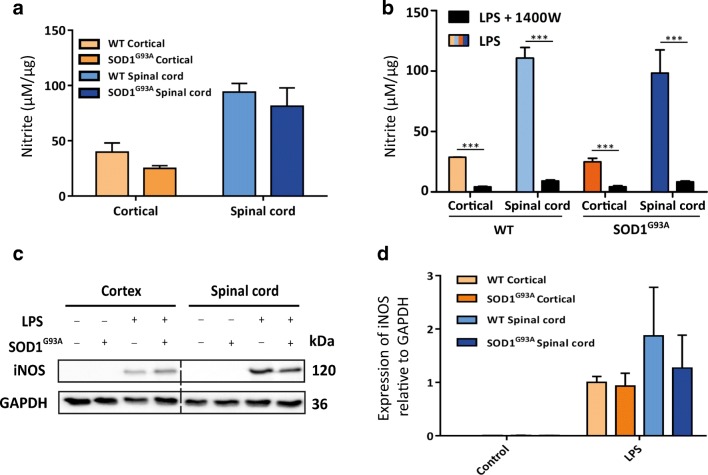
SOD1^G93A^ and WT glia produce similar levels of NO and iNOS in response to inflammatory stimuli. **a** Nitrite/nitrate production of WT and SOD1^G93A^ glia following treatment with 80 μg/ml LPS and/or 1400W (**b**). **c** Immunoblot for iNOS expression in WT and SOD1^G93A^ mixed glial cultures. **d** Quantification of immunoblots for iNOS expression in WT and SOD1^G93A^ mixed glial cultures. Error bars = S.E.M.

Glial cells are known to play a key role in determining the extent of motor neuron death in ALS (Boillee et al. 2006; Yamanaka et al. 2008). In order to assess whether the increased reactivity of spinal cord glia to pro-inflammatory stimuli observed in the previous experiments may contribute to the greater neuronal loss in the spinal cord of SOD1^G93A^ mice compared to the cortex, we next investigated the induction of the NFκ-B-mediated inflammatory pathway in primary mixed glial cultures derived from SOD1^G93A^ mice. Although spinal cord mixed glial cultures demonstrated significantly higher concentrations of NO in both WT and SOD1^G93A^ cultures compared with corresponding cortical cultures, there were no differences in either NO production or iNOS expression between WT and SOD1^G93A^ cultures from either region (Fig. 3). These findings suggest that the expression of SOD1^G93A^ in cortical and spinal cord glial in itself does not exacerbate NO production nor induce detectable iNOS expression in response to inflammatory stimuli.

### Cortical and spinal cord glia elicit a similar heat shock response

Glial cells provide protection to neurons in diverse ways, including through elevation of the HSR under conditions of stress. Therefore, we next studied the ability of glia to induce the HSR after exposure to inflammatory stimuli and heat shock, and examined whether there were differences in the HSR of spinal and cortical glia.

**Fig. 4:**
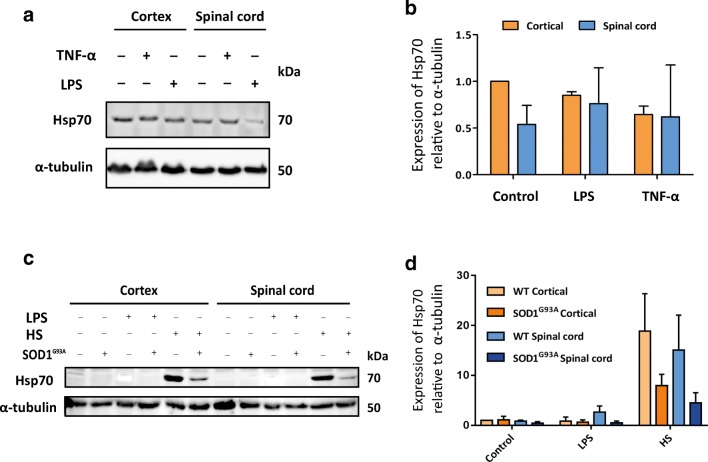
Hsp70 upregulation is impaired following heat stress in SOD1^G93A^ cortical and spinal cord glial cultures. **a** Immunoblot for Hsp70 in cortical and spinal cord glia treated with 80 μg/ml LPS or 100 ng/ml TNF-ɑ. **b** Quantification of immunoblot for Hsp70 in cortical and spinal cord glia treated with 80 μg/ml LPS or 100 ng/ml TNF-ɑ. **c** Immunoblot for Hsp70 in WT and SOD1^G93A^ cortical and spinal cord mixed glial cultures treated with 80 μg/ml LPS or heat shock of 42 °C for 30 min. **d** Quantification of immunoblot for Hsp70 in WT and SOD1^G93A^ cortical and spinal cord mixed glial cultures treated with 80 μg/ml LPS or heat shock of 42 °C for 30 min. Error bars = S.E.M.

As shown in Fig. 4 a and b, exposure to inflammatory stimuli (LPS and TNF-ɑ) did not cause a significant HSR in either spinal or cortical wild-type glial cells. However, following heat shock, there was a significant HSR in both cortical- and spinal cord–derived glial cultures, with a clear increase in expression in Hsp70, which was upregulated to a similar extent in spinal cord and cortical cultures (Fig. 4c, d).

### Expression of ALS-causing SOD1^G93A^ impairs Hsp70 upregulation in glia following heat stress

We next compared the level of heat shock–induced upregulation of Hsp70 between WT glia and glia expressing mutant SOD1^G93A^. As can be seen in Fig. 4c, d, the HSR of glia expressing SOD1^G93A^ was reduced compared to WT glia, so that following exposure to heat stress, the increase in Hsp70 expression was greater in WT than in SOD1^G93A^ glial cultures. This suggests that the expression of ALS-causing mutant SOD1^G93A^ induces a deficit in the cytoprotective HSR in SOD1^G93A^ glia. Interestingly, this deficit in stress-induced Hsp70 induction was present in both cortical and spinal cord glial cultures expressing the SOD1^G93A^ mutation.

Taken together, our results show that spinal cord glia display a greater inflammatory response following exposure to inflammatory stimuli than cortical glia, and this differential stress response is not altered by the expression of mutant SOD1^G93A^. In contrast, the HSR of glia is not influenced by the CNS region of origin, but is diminished in SOD1^G93A^ expressing glia from both the cortex and the spinal cord.

### Heat stress reduces the activation of NF-κB signalling and iNOS expression in spinal cord glia

Previous studies have shown that there is crosstalk between the HSR and the NF-κB-mediated inflammatory pathway (Chen et al. 2006; Ran et al. 2004). Heat stress reduces iNOS activity and mRNA levels induced by LPS treatment in cortical astroglial cultures (Feinstein et al. 1996). Therefore, we next investigated whether there is a relationship between the protective HSR and the pro-inflammatory NF-κB-mediated inflammatory pathway in the spinal cord and cortical glia. We combined LPS and heat shock treatments to determine whether induction of the HSR regulates the activation of the NF-κB-mediated inflammatory pathway in cortical- and spinal cord–derived mixed glial cultures.

**Fig. 5:**
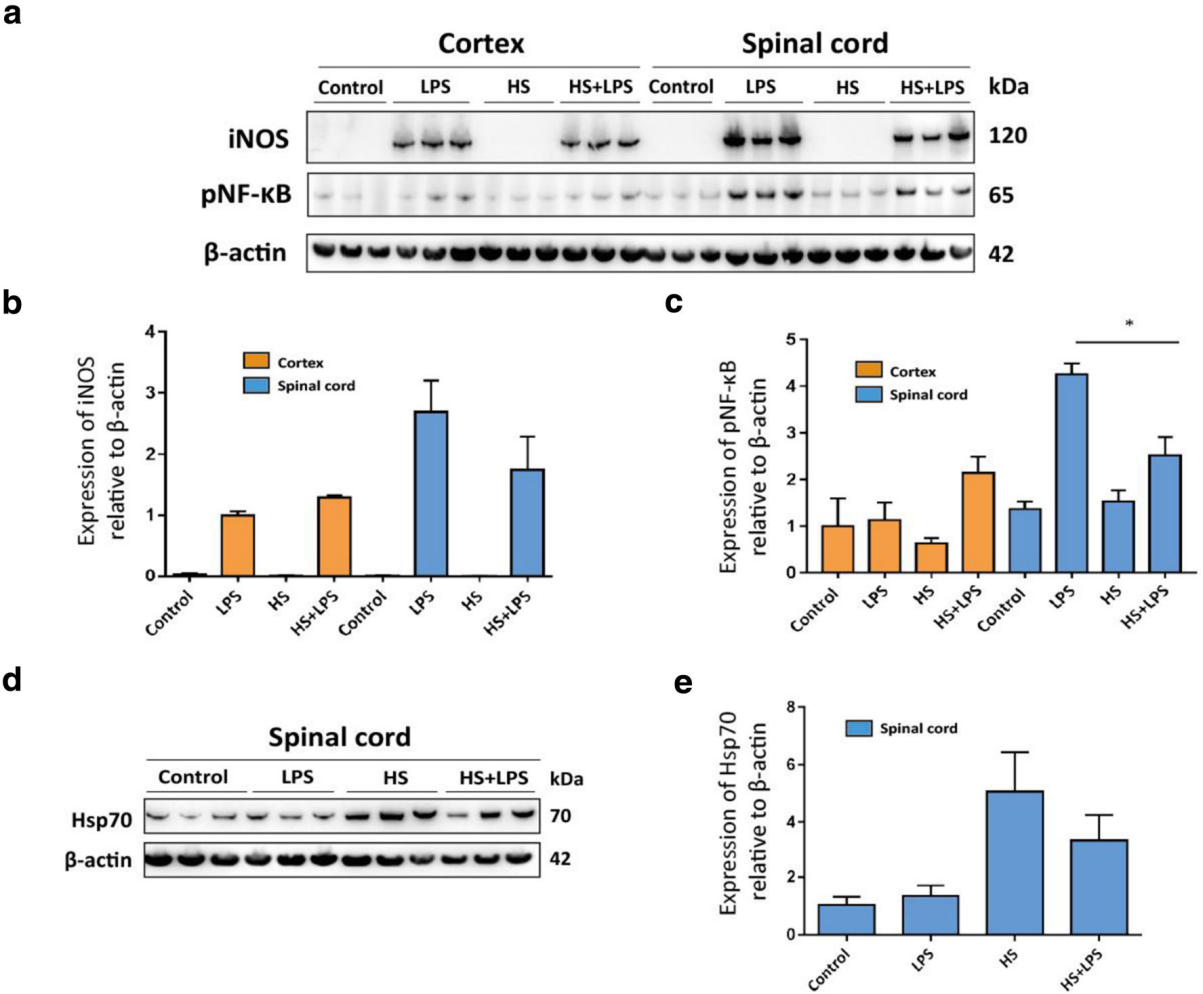
Heat stress reduces levels of LPS-induced iNOS and pNF-κB in spinal cord mixed glial cultures. **a** Immunoblot for iNOS and pNF-κB expression following treatment with heat shock at 42 °C for 30 min and/or 80 μg/ml LPS. Quantification of immunoblots for iNOS (**b**) and pNF-κB (**c**) following treatment with heat shock at 42 °C for 30 min and/or 80 μg/ml LPS. **d** Immunoblot for Hsp70 expression following treatment with heat shock at 42 °C for 30 min and/or 80 μg/ml LPS. **e** Quantification of immunoblots for Hsp70 following treatment with heat shock at 42 °C for 30 min and/or 80 μg/ml LPS. Error bars = S.E.M.

The results summarised in Fig. 5 show that heat shock limited the increase of iNOS expression in spinal cord glia observed following treatment with LPS (Fig. 5a, b). Phosphorylation of the NF-κB complex, the upstream effector of iNOS, is required for its transcriptional activity (Hayden and Ghosh 2008). In line with decreased iNOS expression, heat stress caused an attenuation of LPS-induced phosphorylation of the NF-κB complex in spinal cord glia, but not cortical glia (Fig. 5a, c). Increased Hsp70 expression following heat stress was unaffected by the addition of LPS in spinal cord glia (Fig. 5d, e).

Furthermore, although treatment of spinal cord glia with inflammatory stimuli induced a clear increase in iNOS levels, this effect was reduced by heat stress (Fig. 5). The inhibitory effect of heat stress on the induction of iNOS may be mediated by a heat shock–induced increase in Hsp70, which is known to limit activation of NF-κB (Chen et al. 2006) (Fig. 5c). Compared to cells treated with LPS alone, spinal cord glia exposed to both heat stress and LPS displayed a reduced activation of NF-κB, as determined by the levels of phosphorylated NF-κB.

## Discussion

The abundance of glial cells and the support they provide to different neuronal populations varies across different regions of the CNS. In this study, we explored whether regional differences between cortical- and spinal cord–derived glia exist in terms of their response to inflammatory stimuli and activation of the HSR. Activation of the NF-κB-mediated inflammatory pathway in glial cells is thought to contribute to an accelerated disease progression in neurodegenerative diseases such as ALS and, therefore, differences in this pathway might hold important clues for the selective vulnerability of different neuronal cell populations in disease. The results of this study show that, compared to cortical glia, glia from the spinal cord were more prone to inflammatory stimuli and produced elevated levels of iNOS, which resulted in increased NO release. Interestingly, expression of cytoprotective Hsp70 was not augmented in spinal cord cultures compared to cortical glia in response to heat stress or inflammatory stimuli, suggesting that the magnitude of the HSR is similar in cortical and spinal cord glia.

We also examined glial cultures obtained from mice overexpressing the ALS causing SOD1^G93A^ mutation to reveal any disease-relevant alterations in inflammatory responses and the HSR in different glial populations. Interestingly, we found that although the inflammatory response in SOD1^G93A^ expressing glia was the same as in wild-type glia from corresponding regions, SOD1^G93A^ expressing glia had a significant impairment in the ability to upregulate Hsp70 in response to stress.

Activation of NF-κB drives iNOS expression and subsequent NO production, which has been shown to play a role in the pathomechanisms of ALS (Yuste et al. 2015). Knockout or pharmacological inhibition of iNOS using 1400W extends the lifespan of SOD1^G93A^ mice (Martin et al. 2007; Chen et al. 2010). Furthermore, blocking NO production with 1400W has also been shown to be beneficial in several other pathological conditions, reducing aggregate formation in a model of ischaemia (Chen et al. 2012) and traumatic brain injury (Jafarian-Tehrani et al. 2005), and reducing pain hypersensitivity in a model of neuropathic pain (Staunton et al. 2018).

While activation of NF-κB, the upstream effector of iNOS, is well established in animal models and human ALS (Casciati et al. 2002; Migheli et al. 1997; Sako et al. 2012; Xu et al. 2018), the involvement of the various cellular components of NF-κB activation and its role in disease is ambiguous. NF-κB activation has been shown to be detrimental to motor neuron survival (Ikiz et al. 2015), although NF-κB deficiency in motor neurons had also been reported to cause motor neuron death in ALS (Sulejczak et al. 2015). With respect to the role of NF-κB in glial cells, some reports suggest that microglia are the key cellular components regulating neuroinflammation through NF-κB activation (Frakes et al. 2014), whereas astroglial NF-κB activation has no role in disease (Crosio et al. 2011). A more subtle, regulatory role has been proposed for astroglial NF-κB activation, in which astroglial NF-κB regulates inflammatory processes differently at different stages of the inflammatory pathway (Ouali Alami et al. 2018). Previous reports have also suggested a dual role for astroglial NF-κB in ALS, whereby complete abolition of this important master regulator is as detrimental as its over-activation (Lee et al. 2006).

An important aspect of glial function is to respond to environmental and physiological stressors in order to protect the viability of neurons. In post-mortem tissues of ALS patients, there have been reports of reduced Hsp70 expression, while there is also evidence for Hsp70 accumulating in intracellular aggregates in the spinal cord (Bruening et al. 1999; Watanabe et al. 2001), which in turn creates a deficit of cytosolic Hsps (Koyama et al. 2006; Zetterstrom et al. 2011; Okado-Matsumoto and Fridovich 2002). Depletion of cytosolic Hsps is a phenomenon not only exclusively present in the diseased CNS, but has also been described in aged tissues, including in astroglia (Boisvert et al. 2018). It has been proposed that age-dependent failure of the HSR contributes to the development of neurodegenerative conditions and therefore HSR dysfunction may be part of the primary pathology of neurodegenerative diseases including ALS (Calderwood and Murshid 2017). Thus, increasing Hsp levels in neurons and glial cells has been investigated as an attractive therapeutic target for protein misfolding diseases such as ALS (Kalmar et al. 2014; Kieran et al. 2004; Waza et al. 2005). The HSR and its central effector, Hsp70, also play an important role in other cellular processes implicated in ALS, including stress granule formation, autophagy and proteasomal degradation, as well as inflammatory signalling (Kalmar and Greensmith 2017; Luders et al. 2000; Walters and Parker 2015; Kon and Cuervo 2010; Kabashi et al. 2004; Yost and Lindquist 1991; Osaka et al. 2016; Ganassi et al. 2016).

It is possible that intrinsic differences in these stress responses of glia from different regions of the CNS could contribute to the vulnerability of specific neuronal groups to specific stressors, leading for example to the selective death of motor neurons in ALS, or dopaminergic neurons in Parkinson’s disease. In this study, Hsp70 expression was used as a marker for activation of the HSR as this major Hsp is the most responsive to environmental stressors (Sharp et al. 2001; Nollen and Morimoto 2002). However, we did not observe any intrinsic regional differences between the stress-induced expression of Hsp70 in spinal cord and cortical glia. Interestingly, there was an impairment of Hsp70 induction in response to heat stress in both cortical and spinal cord glia expressing SOD1^G93A^. Thus, although there were no region-specific differences in Hsp70 expression between cortical and spinal cord glia, SOD1^G93A^ glia from both regions displayed an impaired Hsp70 induction in response to stress compared to WT glia.

We also investigated the possibility that the NF-κB-mediated inflammatory pathway and the HSR may interact with each other in a region-specific manner. The protective effects of heat shock in neuroinflammatory and neuronal stress conditions are well characterised (Murphy et al. 2002; Zheng et al. 2008; Gifondorwa et al. 2007). This protective effect is at least partly attributed to the effects of elevated Hsp70 levels inhibiting iNOS activation (Feinstein et al. 1996). Indeed, it has been shown that pharmaceutical upregulation of Hsp70 can lead to increased resistance to pro-inflammatory stimuli in glial cells (Kacimi and Yenari 2015). In view of the negative regulation of NF-κB by Hsp70 to stimuli in spinal cord glia of WT and SOD1^G93A^ cultures, we hypothesise that impaired Hsp70 induction could contribute to an exacerbated inflammatory response in ALS. Our results show that in spinal cord–derived glial cells exposed to a combination of heat stress and inflammatory stimuli, expression of iNOS and activation of NF-κB was indeed lower than in glia exposed to inflammatory stimuli alone. Interestingly, in our experiments, we did not observe a cumulative effect of these two stressors on the expression of Hsp70 and thus, it appears that the heat shock–induced activation of the HSR is sufficient to limit the inflammatory pathway. Therefore, elevated levels of Hsp70 can limit the activation of iNOS expression and potentially achieve therapeutic benefits.

## Conclusions

The results of this study show that spinal cord–derived glia are intrinsically more prone to activation of the NF-κB-mediated inflammatory pathway and iNOS induction than glia derived from the cortex, which may contribute to the selective vulnerability of spinal cord motor neurons in SOD1^G93A^ ALS mice. Expression of SOD1^G93A^ limited the induction of the HSR, resulting in the removal of the negative regulation of NF-κB activation by Hsp70. Our results suggest that since anti-inflammatory approaches alone have not proved successful in ALS, a more effective therapeutic strategy to combat motor neuron death in ALS should involve intervention at multiple levels, for example, strengthening the intrinsic stress responses while also limiting inflammation.

## Experimental procedures

### Breeding and maintenance of SOD1^G93A^ and wild-type mice

All experiments were performed in accordance with the Animals (Scientific Procedures) Act 1986 and following approval from the Institute of Neurology’s Animal Welfare and Ethical Review Board. Mice were housed in individually ventilated cages on a 12-h light/dark cycle with food and water made available ad libitum. SOD1^G93A^ (B6SJL-Tg(SOD1*G93A) 1Gur/J), overexpressing human mutant SOD1^G93A^ (glycine to alanine substitution at position 93), were originally obtained from Jackson Laboratories (USA) along with wild-type (WT) littermates. Mice were maintained by breeding heterozygous male carriers with female (C57BL/6 × SJL) F1 hybrids. WT mice used in this study were obtained from the same colony and thus had the same genetic background as the SOD1^G93A^ mouse colony. Tail biopsies were genotyped for expression of mutant SOD1^G93A^ by PCR amplification of genomic DNA, analysed by electrophoresis using a 2% agarose (Sigma-Aldrich) gel stained with GelRed (Sigma-Aldrich) and visualised using a ChemiDoc imager (Bio-Rad).

### Primary mixed cortical and spinal cord glial cultures

Primary mixed glial cultures were obtained from SOD1^G93A^ and WT mice at postnatal day 2–3. Mice were humanely killed by decapitation and spinal cords and cortices were dissected, meninges removed and cut into small 1 mm × 1 mm pieces. Tissue was then digested in 0.025% trypsin, 0.017% (*w*/*v*) DNase I (Sigma-Aldrich), 0.3% (*w*/*v*) Bovine Serum Albumin (BSA) and 1% penicillin/streptomycin in Hanks Balanced Salt Solution (HBSS) (Thermo Fisher) for 10 min at 37 °C. Foetal bovine serum (FBS) (Thermo Fisher) was used to inhibit the proteolytic reaction. Tissue was triturated 10–15 times and then centrifuged at 1000×*g* for 5 min. The resulting cell pellet was resuspended in feeding media containing 15% FBS and 1% penicillin/streptomycin in DMEM supplemented with 2 mM Glutamax (Thermo Fisher) and filtered through a 100-μm nylon strainer before being seeded on plates coated with 10 μg/ml poly-D-lysine. Cells were maintained under standard culture conditions (37 °C and 5% CO_2_) and media were replenished every 3 days.

### Treatment of primary mixed glial cultures

After being maintained for 12 days in vitro, when primary cortical and spinal cord mixed glial cultures are confluent and at a quiescent state, without substantial presence of microglial proliferation, cells were treated with either of the inflammatory inducers: tumour necrosis factor-alpha (TNF-ɑ) (10–200 ng/ml) or lipopolysaccharide (LPS) (2–100 μg/ml) for 24 h, or heat shocked at 42 °C for 30 min and allowed to recover at 37 °C for 24 h. In instances where cultures were treated with both LPS and heat shocked, cultures were treated with LPS, immediately heat shocked at 42 °C for 30 min and then incubated at 37 °C for 24 h. In some instances, cells were also pre-treated for 2 h with 1400W, a specific iNOS inhibitor, used at 25 μM. Cells were subsequently processed for immunoblotting, immunostaining or flow cytometric analysis at various timepoints but in most cases, if not mentioned, 24 h later.

### Immunofluorescence staining

Primary mixed glial cultures were fixed in 4% paraformaldehyde (PFA) in phosphate buffered saline (PBS) for 10 min and then washed with PBS. Cells were then blocked for 1 h at room temperature (RT) in blocking solution consisting of 5% normal donkey serum in PBS containing 0.1% Triton X-100 (PBST). Primary antibodies rabbit anti-GFAP 1:10,000 (abcam ab7260) and rabbit anti-Iba-1 1:100 (Wako LKR1186) were then added in blocking solution overnight at 4 °C. The following day, cells were washed with PBS and then incubated in secondary antibodies (anti-rabbit or anti-mouse Alexa Fluor® 488 or Alexa Fluor® 596 raised in donkey, by Thermo Fisher, used at 1:1000) in blocking solution for 2 h at RT. After another set of PBS washes, a DAPI stain was applied (Sigma-Aldrich, 1:2000) to stain nuclei and then cells were mounted with Mowiol mounting media and stored at 4 °C. Imaging was performed using a Leica inverted epifluorescence light microscope and Leica Application Suite software. The proportion of cells that were GFAP^+ve^ or Iba1^+ve^ were determined using MetaMorph Image Analysis Software (Molecular Devices, CA, USA) by using the number of DAPI-labelled nuclei as the total number of cells in the culture.

### Immunoblotting

Primary mixed glial cultures were washed with PBS and then lysed on ice with RIPA buffer (50 mM Tris pH 7.5, 150 mM NaCl, 1% NP40, 0.5% sodium deoxycholate, 1 mM EGTA, 1 mM EDTA and protease and phosphatase inhibitors (Halt™ Protease and phosphatase Inhibitor Cocktail, Thermo Fisher)). Protein content of samples from primary mixed glial cultures was estimated by protein assay (Bio-Rad DC protein assay) according to the manufacturer’s instructions with absorbance at 750 nm measured using a FLUOstar Omega microplate reader (BMG LabTech). Samples were subsequently diluted to equal concentrations in RIPA buffer and then added 4:1 in sample buffer (Laemmli buffer supplemented with 10% β-mercaptoethanol) and heated at 95 °C for 10 min to denature proteins. Protein samples were loaded onto precast 4–12% NuPage Bis-Tris gels (Thermo Fisher) and run in NuPage MES SDS running buffer (Thermo Fisher) at 160 V for 1 h. Protein was then transferred onto nitrocellulose membranes (Amersham Biosciences) at 100 V for 1 h in transfer buffer (National Diagnostics) containing 20% methanol. A Ponceau S stain (Sigma-Aldrich) was used to confirm efficient protein transfer before membranes were blocked in 5% BSA (VWR) in Tris buffered saline with 0.1% Tween 20 (TBST) for 1 h. Membranes were then probed with primary antibodies mouse anti-Hsp70 1:1000 (SantaCruz W27: Sc-24), rabbit anti-iNOS 1:1000 (abcam ab178945), rabbit anti-phospho-NF-κB (p65) (Ser536) 1:1000 (Cell Signalling 93H1: #3033), mouse anti-GAPDH 1:1000 (Millipore AB2302), mouse anti-ɑ-tubulin 1:1000 (Thermo Fisher 236-10501) and mouse anti-β-actin 1:40,000 (abcam ab8226) diluted in blocking solution and left on a shaker overnight at 4 °C. The next day membranes were washed in TBST and then incubated with secondary antibody (anti-mouse or anti-rabbit Ig conjugated to horseradish peroxidase (Dako), used at 1:5000) for 2 h at RT. After another set of TBST washes, proteins were visualised using Luminata Crescendo chemiluminescence reagent (Millipore). Images were taken using a ChemiDoc imager (Bio-Rad) and bands were quantified using ImageLab software (Bio-Rad). Relative changes in protein expression were measured and normalised to housekeeping proteins β-actin, ɑ-tubulin or GAPDH.

### Flow cytometry

Primary mixed glial cultures were suspended using 0.025% trypsin and washed once with PBS. Cells were fixed in 4% (*w*/*v*) paraformaldehyde for 15 min in suspension, washed twice in PBS and then permeabilised and blocked in 5% (*v*/*v*) normal goat or donkey serum (or BSA) PBST for 1 h at RT. Cells were then incubated with primary antibodies mouse anti-GFAP conjugated to Cy3 1:1000 (Sigma-Aldrich), and mouse anti-CD11b antibody conjugated to Cy7 1:1000 (Tonbo Biosciences) in 3% BSA in PBS for 2 h at RT and then washed three times in PBS. The entire immunolabelling process was conducted with gentle rocking at each step to ensure appropriate mixing of cells with fixing, permeabilising, blocking and antibody solutions. Cells were then centrifuged at 400×*g* for 5 min and resuspended in PBS before being analysed using a FACS Aria II equipped with a 488 nm, 561 nm and 633 nm lasers. A minimum of 20,000 events per sample was collected at a high flow rate. Forward scatter was collected using a linear scale and side scatter in log scale, and fluorescent emissions were collected as area (log scale) for each channel. For Cy3 fluorescence, data were collected with the 561 nm laser and 582/15 bandpass filter. For Cy7 fluorescence, data were collected with the 633 nm laser and the 670 longpass filter.

### Nitric oxide production measurements

Release of nitric oxide from primary mixed glial cultures following exposure to inducers of inflammatory signalling was indirectly measured using a Griess assay. Media from cultures were collected 24 h after treatment and nitrates in the culture were converted to nitrite by incubating media with nitrate reductase (0.028 U/ml) and β-NADPH (100 μM) for 15 min at 37 °C*.* Media were then mixed in a 1:1 ratio with modified Griess reagent (Sigma-Aldrich). Sodium nitrite standards (NaNO_2_) were prepared in feeding media and absorbance measured using a spectrophotometer at 540 nm. Total protein levels were then measured (Bio-Rad DC protein assay) using a spectrophotometer at 750 nm and nitrite levels were normalised accordingly.

### ELISA

GFAP expression in cortical and spinal cord mixed glial cultures were compared quantitatively using a GFAP ELISA (Petzold et al. 2004). Briefly, a 96-well flat bottom plate (Maxisorb NUNC, VWR) was coated overnight at 4 °C with the capture antibody, mouse anti-GFAP (1:1000), in 0.05 M carbonate buffer, pH 9.5. The plate was washed in barbitone buffer (14.22 μM sodium barbitone, 2.28 μM barbital, 0.24 μM EDTA) supplemented with 0.2% BSA and 0.05% Tween 20. After washing, 50 μL of sample diluent (barbitone buffer supplemented with 6 mM EDTA, and 0.2% BSA) and 50 μL of GFAP standards or whole cell lysates from mixed glial cultures were then added in quadruplicate to the plate. The plate was incubated for 1 h at RT. After washing with barbitone buffer, the secondary antibody (bovine anti-GFAP antibody 1:1000) was diluted in barbitone buffer, added to each well and incubated for 1 h at RT. After washing, the tertiary antibody (HRP-conjugated swine anti-rabbit IgG 1:1000) was diluted in barbitone buffer and added to each well. The plate was then incubated with supersensitive 3, 3′, 5, 5′ tetramethylbenzidine liquid substrate (Sigma-Aldrich) for 30 min at RT in the dark. The reaction was stopped by adding 1 M HCl and the absorbance was read spectrophotometrically at 450 nm, with 750 nm as the reference wavelength on a Wallac Victor 2 plate reader. Concentration of GFAP content in the samples was then calculated by comparing absorbance values to absorbance values measured from a standard curve containing samples of known concentrations of GFAP.

### Statistical analysis

Results are presented as the mean ± SEM for at least three biological repeats (three litters of newborn mice). Differences between the means were assessed by ANOVA with post hoc comparisons tests or Mann-Whitney *U* test when appropriate using GraphPad Prism Version 7.0 software. Statistical significance was set at *p* = 0.05. Significance is indicated in the figures where appropriate and depicted as: *p < 0.05 **p < 0.01 ***p < 0.001. Scale bar 20 μm.

## Abbreviations and nomenclature

ALS: amyotrophic lateral sclerosis
HSP: heat shock protein
HSR: heat shock response
iNOS: inducible nitric oxide synthase
NF-κB: nuclear factor kappa B
SOD1: superoxide dismutase-1
